# Contamination of Clams with Human Norovirus and a Novel Hepatitis A Virus in Cameroon

**DOI:** 10.1007/s12560-020-09432-2

**Published:** 2020-06-27

**Authors:** Patrice Bonny, Marion Desdouits, Julien Schaeffer, Pascal Garry, Jean Justin Essia Ngang, Françoise S. Le Guyader

**Affiliations:** 1 Laboratoire de Microbiologie, LSEM/SG2M, IFREMER, 44300 Nantes, France; 2 Département de Microbiologie, Université de Yaoundé I, B.P. 812, Yaoundé, Cameroun; 3 Centre de Recherche en Alimentation et Nutrition, IMPM, B.P. 6163, Yaoundé, Cameroun; 4 Laboratoire de Microbiologie, LSEM/SG2M, IFREMER, BP 21105, 44311 Nantes Cedex 03, France

**Keywords:** Shellfish, Hepatitis A virus, Molecular epidemiology

## Abstract

Shellfish constitute an important protein source but may be contaminated by viruses from various origins. A study performed on clams collected in Cameroon showed a high prevalence of norovirus and hepatitis A virus. After sequencing, the hepatitis A virus showed similarities with the genotype V simian strains.

The need for a one-health approach to protect humans from emerging diseases is evident by the high number of microbial pathogens that become zoonotic following insect bites or consumption of contaminated meat. This is particularly well documented for infectious disease transmission from non-human primates to human (Devaux et al. 2019). Environmental issues are critical in such events, especially for RNA viruses that may be excreted at high concentrations by infected hosts and are very resistant outside their hosts (De Graaf et al. 2017). Transmission can occur directly by ingestion of contaminated waters or via contaminated food. For example, shellfish growing in coastal areas or rivers are known to concentrate microorganisms by their ability to filter large volume of waters and thus may favor the transmission of zoonotic strains to humans when consumed.

The demand for shellfish is increasing worldwide given their nutritive value and taste. In addition, shellfish grow without human intervention, are easily collected and constitute an important source of nutrients for many populations (Venugopal and Gopakumar 2017). In West-Africa, the Sanaga clams *Egeria radiata*, a freshwater clam, grow in several rivers, and constitute a substantial source of protein for the local population as well as an important economic income through the shell industry, such as the ceramic or animal feed processing industries (Ajonina et al. 2005). Up to now, no study has been conducted to evaluate the microbial contamination of these clams. Despite being mainly consumed following cooking, which may limit the risk of bacteria transmission, the evaluation of the presence of human enteric viruses implicated in cooked food-borne outbreaks was considered necessary (Pinto et al. 2009; Lunestad et al. 2016).

Fourteen samples of clams (*Egeria radiata*) were collected on the Sanaga river (Cameroon, west Africa, 12 km upstream of the Atlantic Ocean), between February 2018 and March 2019 from two sampling points (eight from site B and six from site M). Clams were immediately frozen and shipped to the Ifremer laboratory where they were dissected and the digestive tissues (DT) recovered. Viruses were eluted from clam DT and nucleic acids were extracted using the reference method (ISO 15216-1 2017). After validation of quality controls (extraction efficiency and inhibitor removal), reverse transcription real-time quantitative PCR was used to detect human norovirus and hepatitis A virus. Human norovirus were detected in ten samples (five from each sampling site) at low concentrations (ranging from below the limit of quantification up to 2500 RNAc/g of digestive tissues for one sample collected in September). Positive samples were amplified using the standard RT-PCR method with the same RT and Platinum *Taq* polymerase enzymes, and primers targeting the polymerase and the capsid regions (Le Guyader et al. 2008). Amplicons from positive samples were purified cloned, sequenced, and genotyped using the Norovirus Typing Tool 2.0 (Kroneman et al. 2011). This approach allowed the identification of norovirus sequences such as GI.2, GII.3, GII.6, GII.4_Sydney 2012 using primers targeting the capsid region or GII.P31, and GII.P21 when using primers targeting the polymerase region. HAV was detected in nine of the samples (four from site B and five from M). The VP1/P2A junction was amplified by PCR, cloned and sequenced, yielding a 441 bp sequence that was identified as HAV and assigned to the genotype V by the HAV Typing Tool (Kroneman et al. 2018). Using NCBI Blastn, the Sanaga strain (GenBank MT185676) displayed a highest similarity (91,88%) with a strain identified in irrigation water samples from South Africa (KZN_Irr-20130530) (Rachida et al. 2016). Bayesian phylogenetic analysis with HAV sequences from different genotypes retrieved from HAVNet and GenBank shows the clustering of these two strains retrieved from environmental samples in a clade supported by high posterior probability and most closely related to simian strains of genotype V (Fig. 1).

**Fig. 1:**
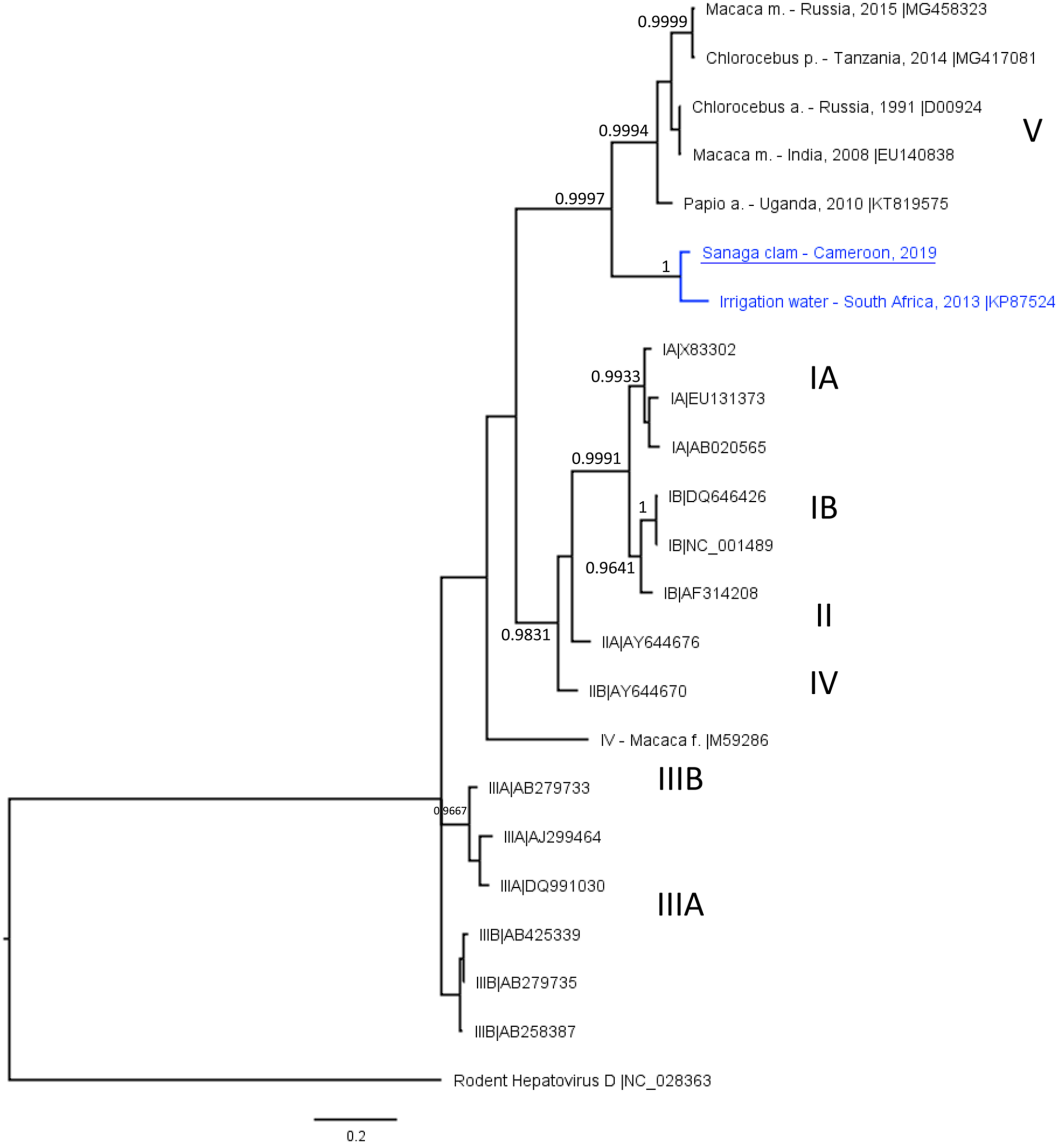
Bayesian phylogeny of HAV sequence identified in the Sanaga clam samples. The tree is based on 441 nucleotides in the VP1-2A junction region (position 2918 to 3359 relative to the HAV reference sequence NC_001489), except for MG417081 (315 nt), MG458323 (346 nt) and M59286 (334 nt) that end before. Sequences were aligned using MUSCLE 3.8.425 implemented in Geneious (11.1.4). The tree was constructed using Mr Bayes 2.3.6 plugin in Geneious, with the GTR evolutionary model, 2 millions generations sampled every 200 steps, and 20% burn-in. Posterior probabilities higher than 0.95 are indicated just above or below the corresponding branches. The rodent hepatovirus D reference sequence was used as an outgroup. HAV sequences cluster by genotype (Gt) as highlighted on the right, with virus from humans belonging to Gt IA-B, IIA-B, and IIIA-B. Simian HAV (host species is indicated in the taxon name) belong to genotype IV or V. HAV from Sanaga clams (underlined) (GenBank MT185676) clusters together with a sequence identified in an irrigation water sample from South Africa, in a clade (blue) separated from simian sequences in the genotype V

The Sanaga strain and KZN_Irr-20130530 exhibited between 79.71 and 82.71% similarities with the simian strains of genotype V, and below 79% with the other genotypes. This suggests, as discussed by Rachida et al., that this clade is too divergent to be considered a subgenotype of genotype V (10). The sequence of the VP3-VP1 cleavage site of KZN_Irr-20130530 suggested a simian origin for this strain (Rachida et al. 2016). The proximity of the Sanaga strain with simian HAV strains and the documented presence of non-human primates in the sampling area suggest a simian origin, but does not exclude another host. Further investigations need to be done to identify human sewage discharge in this area or to compare with clinical cases.

Finding this virus in a food raises several concerns. If the strain originates from simian hosts, the risk of host jump to humans is enhanced by the high frequency of positive samples, the close relation between human and non-human primates, and the high stability of HAV. Indeed, some HAV strains appear highly resistant to heat and cooked coquina clams have been implicated in outbreaks (Pinto et al. 2009). Here this risk appears limited as Sanaga clams are usually consumed boiled or smoked. Nonetheless, the local population needs to be informed of this potential risk of virus transmission (Ajonina et al. 2005). The genomic plasticity of viruses, allowing selection of adaptative mutations may further facilitate their human-to-human transmission (De Graaf et al. 2017). Alternatively, the presence of a new HAV strain in clams also contaminated with human norovirus raises the possibility of a human origin. Further investigations need to be implemented to investigate the origin and prevalence of this new HAV strain in the Sanaga region, and to evaluate the presence of other microbial contaminants in clams and other shellfish.
